# Expression Levels of Obesity-Related Genes Are Associated with Weight Change in Kidney Transplant Recipients

**DOI:** 10.1371/journal.pone.0059962

**Published:** 2013-03-27

**Authors:** Ann Cashion, Ansley Stanfill, Fridtjof Thomas, Lijing Xu, Thomas Sutter, James Eason, Mang Ensell, Ramin Homayouni

**Affiliations:** 1 Department of Acute and Chronic Care, College of Nursing, University of Tennessee Health Science Center, Memphis, Tennessee, United States of America; 2 National Institute of Nursing Research, National Institutes of Health, Bethesda, Maryland, United States of America; 3 Department of Biostatistics and Epidemiology, University of Tennessee Health Science Center, Memphis, Tennessee, United States of America; 4 Department of Biology, University of Memphis, Memphis, Tennessee, United States of America; 5 Methodist University Hospital Transplant Institute, Memphis, Tennessee, United States of America; 6 Transplant Division, Department of Surgery, University of Tennessee Health Science Center, Memphis, Tennessee, United States of America; Universidade de Sao Paulo, Brazil

## Abstract

**Background:**

The aim of this study was to investigate the association of gene expression profiles in subcutaneous adipose tissue with weight change in kidney transplant recipients and to gain insights into the underlying mechanisms of weight gain.

**Methodology/Principal Findings:**

A secondary data analysis was done on a subgroup (n = 26) of existing clinical and gene expression data from a larger prospective longitudinal study examining factors contributing to weight gain in transplant recipients. Measurements taken included adipose tissue gene expression profiles at time of transplant, baseline and six-month weight, and demographic data. Using multivariate linear regression analysis controlled for race and gender, expression levels of 1553 genes were significantly (p<0.05) associated with weight change. Functional analysis using Gene Ontology and Kyoto Encyclopedia of Genes and Genomes classifications identified metabolic pathways that were enriched in this dataset. Furthermore, GeneIndexer literature mining analysis identified a subset of genes that are highly associated with obesity in the literature and Ingenuity pathway analysis revealed several significant gene networks associated with metabolism and endocrine function. Polymorphisms in several of these genes have previously been linked to obesity.

**Conclusions/Significance:**

We have successfully identified a set of molecular pathways that taken together may provide insights into the mechanisms of weight gain in kidney transplant recipients. Future work will be done to determine how these pathways may contribute to weight gain.

## Introduction

Obesity has long been known to be a result of excessive caloric intake and reduced energy expenditure. While some individuals appear able to eat whatever they please with little physical activity and still not gain weight, others gain significant weight. Despite great efforts, researchers have thus far failed at reliably predicting differential weight gain. Genetic variations may be a primary factor. However, identifying a sample that will predictably gain significant weight to test this hypothesis is difficult. Renal transplant recipients are known to gain significant weight (one study reporting an average of 12 kilograms (kg) [1]) during the first year following transplantation. This rapid and large amount of weight gain contributes to the development of cardiovascular disease and other comorbidities, which then leads to less favorable graft outcomes among these individuals [2]. Additionally, the significant variation in weight gain among individuals facilitates determination of unique associations. Many clinicians suggest that the weight gain is related to the prednisone prescribed following transplantation. Interestingly, studies have indicated that prednisone is not the primary cause of weight gain, and that steroid-free protocols alone do not reduce the risk of obesity [3], [4]. Other clinicians suggest that dietary intake may contribute to weight gain. A study done on 44 individuals from the same transplant center found that 96% had a mean caloric intake well under the recommended daily value of 2000 kcal/day, although mean weight increased by more than 10 pounds (4.5 kg) during the six months of the study [5].

Few studies have been conducted using microarray-based gene expression to explore obesity in human subjects and even fewer of these studies have a longitudinal component. Most of the previous work has compared expression levels in subcutaneous and visceral adipose tissue [6], [7]. In both tissues, the up-regulated genes in obese subjects have been shown to be mostly related to inflammation, insulin resistance, leptin signaling pathways, and the immune response [6], [7], [8]. While both forms of adipose tissue can be useful in studies of obesity, subcutaneous adipose tissue has a higher adipogenic potential than visceral fat stores [9] and is thus more relevant in a subject population at high likelihood of increasing abdominal fat mass. Subcutaneous adipose tissue is also an ideal candidate for gene expression studies because of its role in endocrine pathways involved in appetite regulation, such as leptin and insulin signaling [10], [11]. Additionally, subcutaneous adipose tissue can be easily obtained from kidney transplant recipients during transplant surgery.

Although there are few microarray gene expression studies related to obesity, there are many genome wide association studies (GWAS) on obesity related phenotypes. Several genes have been associated with weight gain and obesity. The most commonly cited genes related to obesity are the fat mass and obesity associated gene (FTO) [12], [13], mitochondrial carrier homolog 2 (MTCH2) [13], transmembrane protein 18 (TMEM18), glucosamine-6-phosphate deaminase 2 (GNPDA2), brain derived neurotrophic factor (BDNF), neuronal growth regulator 1(NEGR1), SH2B adaptor protein 1(SH2B1), ETS transcription factor 5 (ETV5), and potassium channel tetramerisation domain (KCTD15) [14].

To our knowledge, there are no published microarray studies that examine gene expression in subcutaneous adipose tissue of kidney transplant recipients. The goal of this study was to identify expression profiles associated with weight gain and to explore the underlying molecular mechanisms of weight gain in kidney transplant recipients by gene expression profiling on subcutaneous adipose tissue. This project was done as preliminary, exploratory work to evaluate the feasibility of a larger scale study. Although exploratory, the results have identified a subset of genes that are associated with obesity that could be explored further as potential markers for identifying at-risk groups for significant weight gain or obesity following kidney transplantation.

## Results

### Subjects

As shown in Table 1, subjects gained a mean of 5±5.9 kilograms with some gaining as much as 17.7 kilograms during the first 6 months following kidney transplantation. About 54% (n = 14) of subjects gained more than 5% of their baseline weight at 6 months. While one subject had as much as a 23% weight gain in 6 months, other subjects lost up to 5% of their total body weight. Overall, our subjects gained an average of 6.5% of their total pretransplant body weight in the first 6 months, a result that is fairly consistent with other reports showing one year weight gains between 6 and 11.8 kg [1], [15]. In the current study, a higher percentage (73%, n = 11) of African American subjects gained weight (>5% of baseline) at 6 months post-transplantation compared to Caucasian subjects (27%, n = 3). Seven of the 26 subjects were diagnosed with type 2 diabetes prior to transplantation, one individual was diagnosed with new onset diabetes after transplantation; thus 31% (n = 8) had diabetes at 6 months.

**Table 1:** Subject characteristics (N = 26).

		Baseline		6 months	
Characteristic		Mean (SD) Range	n (%)	Mean (SD) Range	n (%)
Age at start (years)		51.5 (13.7) 19.4–68.2		N/A	
Male			11 (42)		N/A
Caucasian			11 (42)		
	Gained weight >5%		N/A		3 (27)
African American			15 (58)		N/A
	Gained weight >5%		N/A		11 (73)
Weight (kg)		77.4 (16.0) 57.7–117.2		84.4 (17.9) 60.5–118.4	
BMI (kg/m^2^)		26.7 (3.13) 22.3–33.5		28.54 (3.80) 22.3–34.6	
Weight gain (kg)		N/A		5 (5.9) -3.2–17.7	
Hypertension			24 (92)		24 (92)
Diabetes			7 (27)		8 (31)

Abbreviation: BMI, body mass index.

### Gene expression profiling

**Figure 1:**
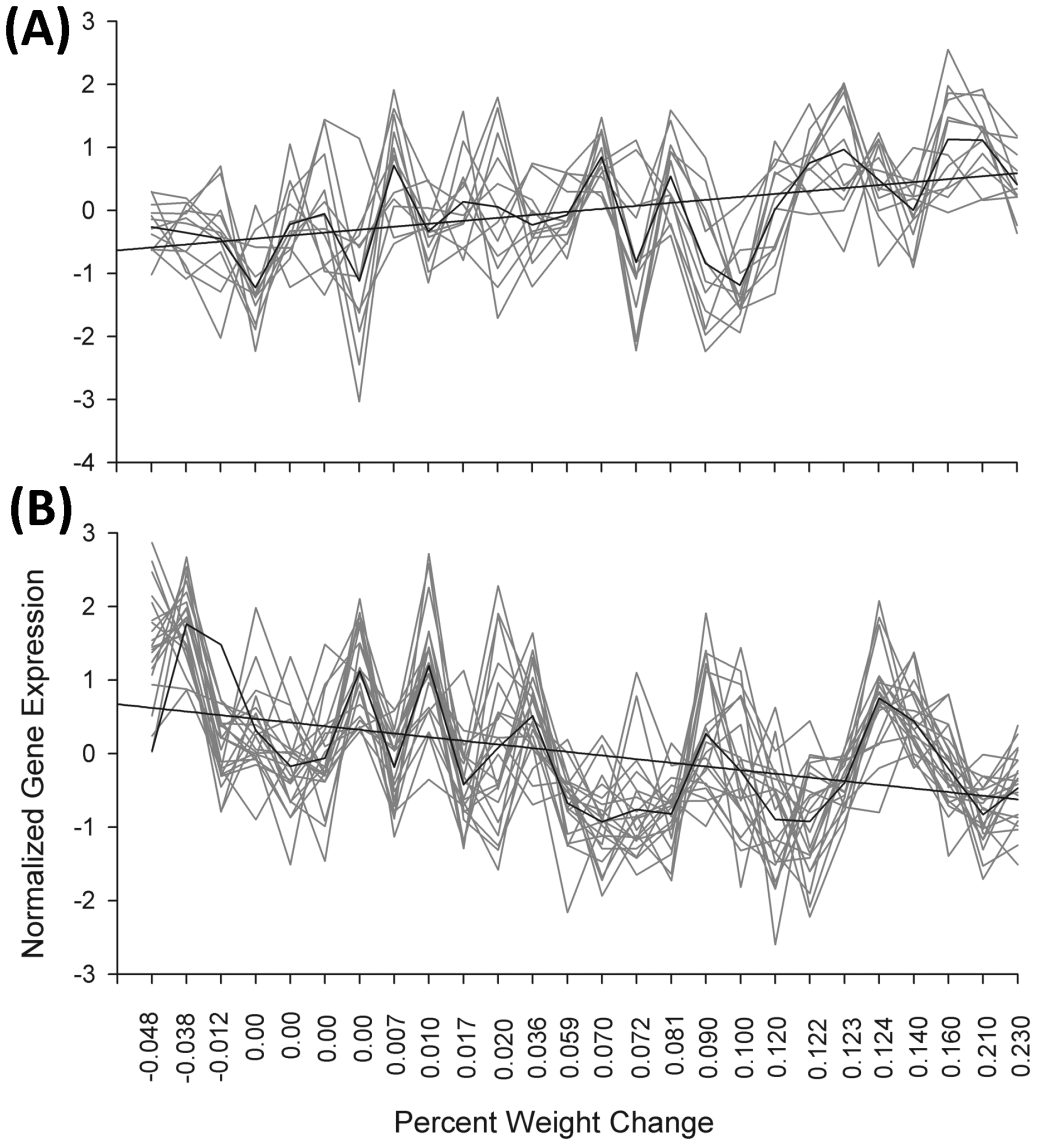
Genes correlated with relative weight change. A set of 12 and 21 obesity related genes that are positively (A) and negatively (B) correlated with relative weight change, respectively. Each line represents the mean normalized expression value (vertical axis) for a given gene across 26 kidney transplant recipients ordered with respect to weight loss/gain (horizontal axis) at 6 months after transplantation.

**Figure 2:**
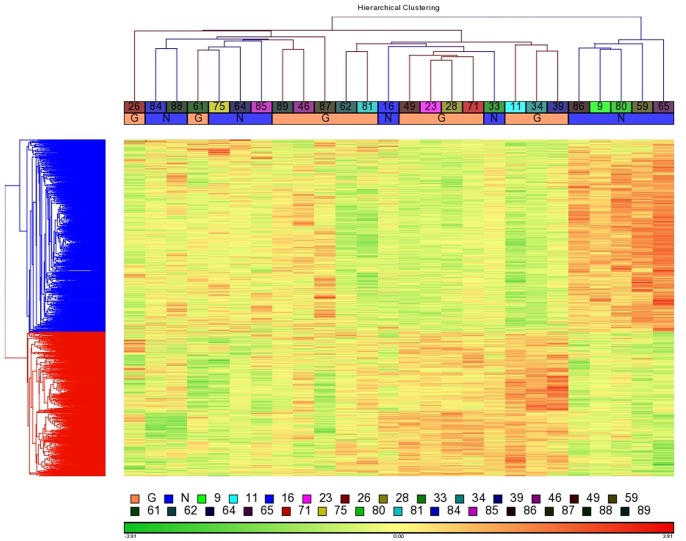
Hierarchical clustering of 1936 genes across 26 kidney transplant recipients. Heatmap representation of expression values for genes (rows) across recipients (columns), whereby low expression is denoted by green and high expression by red. The expression of each gene was normalized across all samples. Recipients were categorized into weight gainers (G, peach color) or non-gainers (G, blue color) if their net weight change was greater or less than 5%, respectively, at 6 months post-transplantation.

**Figure 3:**
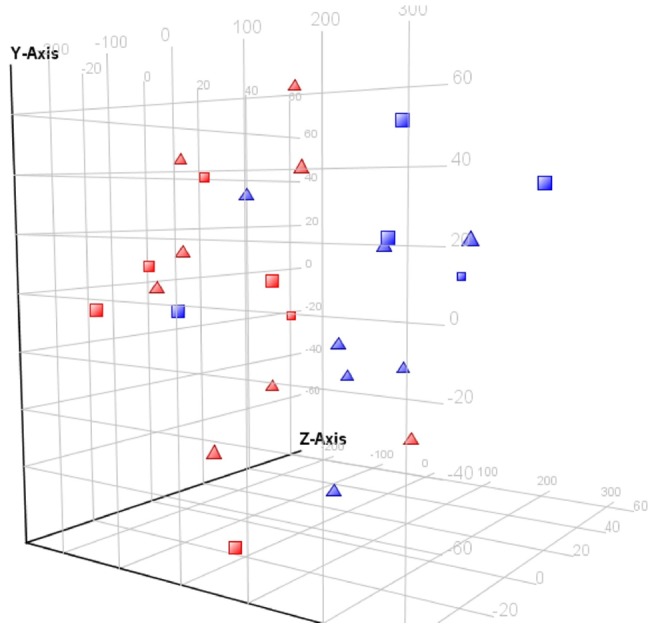
Principle component analysis of 26 transplant recipients using 1936 significantly correlated transcripts with weight change. Approximately 64%, 17% and 10% of the variability was attributed to principle components 1, 2, and 3, respectively. Legend: weight change great >5% at 6 months post-transplantation, *Red*; Weight change <5% at 6 months post-transplantation, *blue*; Male, *square*; Female, *triangle*.

Gene expression levels in adipose tissue of 26 kidney transplant recipients were examined using Affymetrix Human Gene 1.0 ST arrays. Using a regression model that controlled for race and gender, we found 1936 probe targets, corresponding to 1553 unique genes, whose levels were significantly correlated with weight change at 6 months post-transplantation (Tables 2 and 3, Figure 1, and Table S1). It is important to note that there was considerable variability in gene expression across this cohort. This was demonstrated by both hierarchical clustering and principle component analysis on the 1936 probes (Figure 2 and Figure 3). For instance, several non-weight gainer individuals (such as #16 and #33) showed expression profiles similar to weight gainers. Conversely, several weight-gainer individuals (such as #26 and #61) showed expression profiles that were similar to non-gainers based upon hierarchical clustering (Figure 2). Expression variability was not associated with gender or race.

**Table 2:** Expression p-values for genes whose expression levels were positively correlated with weight gain.

Gene ID	Gene Symbol	Gene Title	*p*-value
2888	GRB14	Growth factor receptor bound protein 14	0.00249
1363	CPE	Carboxypeptidase E	0.00467
9563	H6PD	Hexose-6-phosphate dehydrogenase	0.00731
2132	EXT2	Exostoses (multiple) 2	0.0142
80724	ACAD10	Acyl-coenzyme A dehydrogenase family, member 10	0.02042
373156	GSTK1	Glutathione S-transferase kappa 1	0.0235
23452	ANGPTL2	Angiopoietin-like 2	0.02598
284119	PTRF	Polymerase 1 and transcript release factor	0.02863
4482	MSRA	Methionine sulfoxide reductase A	0.02915
9453	GGPS1	Geranylgeranyl Diphosphate synthase 1	0.03223
4889	NPY5R	Neuropeptide Y receptor Y5	0.03482
3952	LEP	Leptin	0.03605
29803	REPIN1	Replication initiator 1	0.0386
5507	PPP1R3C	Protein phosphatase 1, regulatory (inhibitor) subunit 3C	0.03872
55527	FEM1A	Fem-1 homolog a (C. elegans)	0.045
4886	NPY1R	Neuropeptide Y receptor Y1	0.04621
23788	MTCH2	Mitochondrial carrier homolog 2 (C. elegans)	0.04785
1050	CEBPA	CCAAT/enhancer binding protein (C/EBP), alpha	0.04816

**Table 3:** Expression p-values for genes whose expression levels were negatively correlated with weight gain.

Gene ID	Gene Symbol	Gene Title	*p*-value
123722	FSD2	Fibronectin type III and SPRY domain containing 2	0.00481645
374918	IGFL1	IGF-like family member 1	0.00714389
84669	CREB3L3	cAMP responsive element binding protein 3-like 3	0.0081399
54979	HRASLS2	HRAS-like suppressor 2	0.00927293
154	ADRB2	Adrenergic, beta-2, receptor, surface	0.01013697
139760	GPR119	G protein-coupled receptor 119	0.0123002
145264	SERPINA12	Serpin peptidase inhibitor, clade A (alpha-1 antiproteinase, antitrypsin), member 12	0.01684975
5726	TAS2R38	Taste receptor, type 2, member 38	0.01731357
169841	ZNF169	Zinc finger protein 169	0.01758989
2867	FFAR2	Free fatty acid receptor 2	0.01984576
256394	SERPINA11	Serpin peptidase inhibitor, clade A (alpha-1 antiproteinase, antitrypsin), member 11	0.02007965
53630	BCMO1	Beta-carotene 15, 15′-monooxygenase 1	0.02337747
169792	GLIS3	GLIS family zinc finger 3	0.02474613
150379	PNPLA5	Patatin-like phospholipase domain containing 5	0.02854462
1401	CRP	C-reactive protein, pentraxin-related	0.02956737
434	ASIP	Agouti signaling protein, nonagouti homolog (mouse)	0.0311821
2691	GHRH	Growth hormone releasing hormone	0.03308533
619373	MBOAT4	Membrane bound O-acyltransferase domain containing 4	0.03865589
56923	NMUR2	Neuromedin U receptor 2	0.04210583
7021	TFAP2B	Transcription factor AP-2 beta (activating enhancer binding protein 2 beta)	0.04236
55937	APOM	Apolipoprotein M	0.04513979
390259	BSX	Brain specific homeobox	0.04660706
922	CD5L	CD5 molecule-like	0.04777909

To gain insights into the potential mechanism(s) that underlie weight gain, we performed functional analysis on differentially expressed genes using two different bioinformatic approaches. First, we determined which functional classifications in GO or KEGG were enriched in our gene set using a publicly available tool kit called WebGestalt (Table 4 and Table 5). Among the 1553 genes correlated with weight change, GO analysis found 38 genes pertaining to mitochondrial inner membrane (p<0.0002) and a number of genes pertaining to metabolic processes such as oxidative phosphorylation (21 genes, p<0.0003), quinone cofactor metabolism (6 genes, p<0.0002), and oxidoreductase activity (14 genes, p<0.0009) (Table 4). Interestingly, using KEGG analysis (Table 5), we found enrichment of genes associated with a number of neurological diseases such as Parkinson's disease (19 genes, p<0.0013), Alzheimer's disease (23 genes, p<0.0033) and Huntington's disease (22 genes, p<0.0157). Lastly, we found a number of sensory genes represented in our data set. For example, olfactory transduction genes were significantly enriched (44 genes, p<0.0035). GO analysis similarly found that there were 40 genes related to olfactory receptor activity (p = 0.0029), and 44 genes related to sense of smell (p = 0.0008) (Table 4).

**Table 4:** Functional categories in Gene Ontology that were significantly enriched in the genes associated with weight gain.

Gene Ontology category	# of genes	*p*-value
Quinone cofactor metabolic process	6	0.0002
Mitochondrial inner membrane	38	0.0002
Regulation of dopamine secretion genes	5	0.0004
Phosphoprotein binding	8	0.0006
2 iron 2 sulfur cluster binding	6	0.0007
Sensory perception of smell	44	0.0008
Oxidoreductase activity acting on NACH or NADPH	14	0.0009
G protein coupled receptor protein signaling pathway	11	0.0019
Olfactory receptor activity	40	0.0029
Integral to organelle membrane	18	0.0031
Nicotinic acetylcholine-activated cation-selective channel activity	5	0.0037
Acetylglucosamirlytransferase activity	8	0.0053
Neurotransmitter binding	12	0.0086

Notes: Enrichment p-values were calculated using a hypergeometric test for the number of genes expected in each category by chance. Benjamini-Hochberg adjustment was used to correct for multiple testing error.

**Table 5:** Functional categories in KEGG that were significantly enriched in the genes associated with weight gain.

KEGG category	# of genes	*p*-value
Glycosylphosphatidylinositol(GPI)-anchor biosynthesis	8	0.0005
Oxidative phosphorylation	21	0.0003
Parkinson's disease	19	0.0013
Alzheimer's disease	23	0.0033
Olfactory transduction	44	0.0035
Dorso-ventral axis formation	6	0.0102
Huntington's disease	22	0.0157
O-Mannosyl glycan biosynthesis	2	0.0183

Notes: Enrichment p-values were calculated using a hypergeometric test for the number of genes expected in each category by chance. Benjamini-Hochberg adjustment was used to correct for multiple testing error.

In a second approach, we used a commercial literature mining tool called GeneIndexer to investigate the functional relationship of genes to a set of keyword concepts that were not included in GO or KEGG databases. GeneIndexer contains Medline abstracts for 1170 of the 1553 differentially expressed genes. The most relevant query words (key concepts) were chosen by the research team and were refined iteratively by examining the output from GeneIndexer. The literature association scores were used to group genes and keywords by hierarchical clustering. For instance, several conceptually related keywords such as BMI (body mass index), overweight, obesity, adipose, and diabetes were clustered tightly together based on their associated genes in the literature (Table S2 and Figure S1). On the other hand, other keyword concepts such as addiction (chosen based on an emerging association between obesity and food addiction), and dopamine (chosen based on GO/KEGG analysis) were not as closely aligned with obesity concepts in the literature (Figure S1). These results support the validity of GeneIndexer output. We next ranked genes according to the number of associations they had with keyword concepts above a score threshold of 0.1 (Table S2). In GeneIndexer, a score above 0.2 generally suggests an explicit link between the keyword and gene, whereas a score between 0.1 and 0.2 generally suggests an implied relationship between the keyword and gene. We observed three general groups. One group of genes (SERPINA12, CPE, SERPINA11, MTCH2, PNPLA5, INTS1, APOM) was associated (cosine value >0.1) with obesity and diabetes. A second group of genes (CHRNB2, CHRNA4, DRD5, PDYN, and PNOC) was associated with neurological concepts such as dopamine, nicotine, and cognition. Interestingly, a third group of genes (CPE, NPY5R, DRD3, TAS2R38, SLC18A1, G6PC3, NPY1R, and VLDLR) was highly associated with both neurological and obesity concepts.

**Figure 4:**
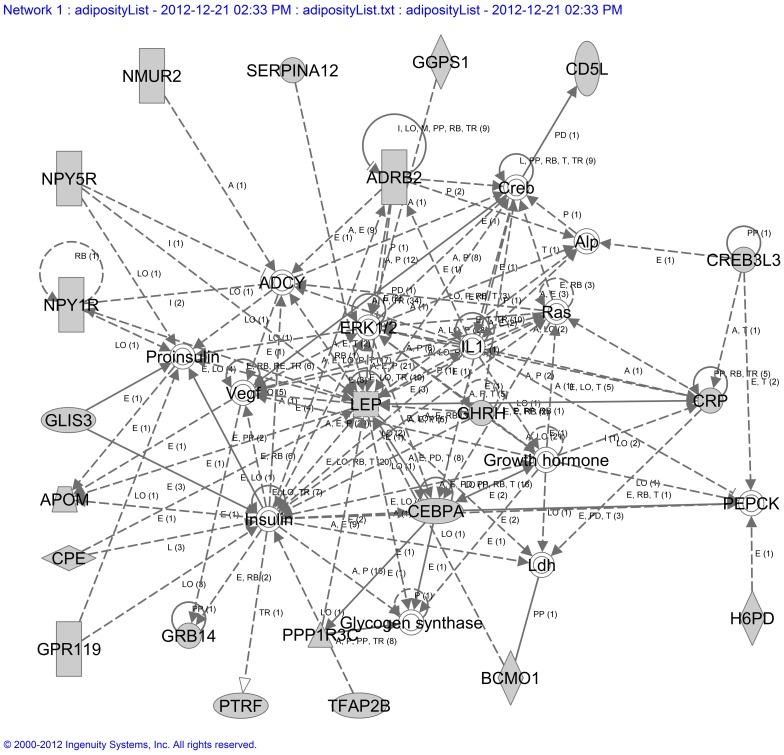
Gene network. Network identified by Ingenuity software from the analysis of the 41 top ranked genes identified to be correlated with weight gain from microarray data and also highly associated with obesity in the literature by GeneIndexer. The genes CPE, NPY1R, NPY5R, and APOM suggest that these subjects may show changes in leptin and insulin response genes prior to post-transplant weight gain.

Next, we examined the molecular interaction networks using Ingenuity Pathway Analysis tool (Ingenuity systems, www.ingenuity.com) for the top 41 genes that were either explicitly or implicitly associated with “obesity” keyword in GeneIndexer. The top ranked network included 22 out of the 41 obesity related genes (Figure 4). Of note, this network shows direct interactions between CPE, APOM, SERPINA12, CRP, NPY1R, and NPY5R genes with leptin (LEP). In general, these and other genes in the network seem to converge on insulin, vascular endothelial growth factor (VEGF), growth hormone, and IL-1 signaling pathways, which are known to play an important role in regulation of metabolism.

## Discussion

### Weight gain in kidney transplant recipients

We, and others, have shown that kidney transplant recipients differentially gain weight after transplantation [15], [16]. Approximately 50% of kidney transplant recipients will gain weight after the surgery [2]. Excessive weight gain after kidney transplant is particularly problematic because the resulting obesity contributes to the development of cardiovascular disease, the primary cause of death in this group [17]. Diabetes is another comorbidity commonly associated with weight gain in this population [18]. To date, a few studies have examined cross-sectional gene expression levels in adipose tissue of obese and non-obese subjects. These studies have identified genes related to adipogenesis [19], as well as glucose and insulin regulation pathways and inflammatory responses [6]. In our longitudinal study, gene expression was examined at baseline and correlated with weight change at 6 months post-transplantation. Our study also found changes in the expression of genes associated with insulin and inflammatory signaling pathways, as well as changes in the expression of leptin. We found a total of 1553 unique genes whose expression levels were significantly correlated with weight change in kidney transplant recipients. Among these, regression analysis found four well-established obesity associated genes that were positively correlated (CPE, LEP, NPY1R, and NPY5R), and two genes (APOM, and CRP) that were negatively correlated with weight gain. Taken together, these results suggest that the individuals in our study may have differences in insulin response genes and leptin signaling prior to transplant, and prior to any post-transplant weight gain. Due to our small sample size, we cannot ascertain whether this result is solely due to some subjects having pretransplant diabetes (31% of the population, or seven individuals), but we suspect that these differences participate in a feed forward process that leads to post-transplant weight gain. Pre-existing differences in insulin and leptin signaling may increase the susceptibility of individuals to gain weight after transplant. Below, we summarize some experimental evidence supporting the potential involvement of specific genes in the feed-forward process of weight gain.

#### CPE

The CPE gene encodes carboxypeptidase E enzyme which is highly expressed in visceral fat deposits in obese subjects [20]. CPE cleaves peptide bonds and is involved in the biosynthesis of several peptide hormones involved in energy balance, including insulin. It also has been suggested that CPE alters proopiomelanocortin (POMC) processing in the hypothalamus, which helps to regulate energy balance in response to circulating leptin and insulin levels [21]. Alterations in any of these signaling pathways can cause increased weight. Indeed, CPE knockout mice have nearly twice the body fat of wild type littermates, have reductions in the breakdown of that fat for energy, and have higher circulating levels of leptin and proinsulin [22].

CPE is also associated with dopamine transporter activity [23]. Dopamine is a neurotransmitter that can play a role in the pleasurable (rewarding) aspects of eating. Thus, not only does CPE play a role in energy balance, it also plays a role in the emotional aspects of eating. From gene ontology analysis, our kidney transplant recipients showed statistically significant increased expression in 5 other genes (p<0.0004) involved in dopaminergic activity (Table 4). Imaging studies have shown that obese subjects may have impairments in dopamine signaling that reduce reward sensitivity [24]. Thus, it is possible that increased expression of the CPE gene could cause alterations in dopamine sensitivity, which in turn causes a decrease in the pleasurable feelings associated with eating. We hypothesize that kidney transplant recipients with enhanced CPE expression may overeat, or choose different foods, in an effort to increase pleasurable feelings associated with eating, which in turn contributes to the weight gain. Support for this idea has been shown in an observational study from the same transplant center [5]. In this study, although nearly all were eating less than 2000 kcal/day, 91% consumed more fat than the daily recommended allowance (35 g), 86% consumed more than the recommended daily carbohydrate allowance (130 g). The study sample as a whole exhibited a mean weight gain of over 10 pounds (4.5 kg) in six months [5].

#### NPY1R and NPY5R

Neuropeptide Y has strong appetite stimulating effects. Continuous administration to the hypothalamus of normal rats has been shown to induce obesity, with reversal of the effects 20 days after discontinuation [25]. In particular, neuropeptide Y receptors 1 and 5 (NPY1R and NPY5R) have been associated with weight gain, and these genes were also found to be highly expressed in the adipose tissue of our transplant recipients. It has been proposed that leptin normally prevents neuropeptide Y from being released from the arcuate nucleus to suppress appetite [26]. If these neuropeptide Y receptors are not sensitive to the effects of leptin, obesity can result [26].

#### APOM

Apolipoprotein M (APOM) is a high density lipoprotein that has been negatively associated with cholesterol level and positively correlated with leptin in humans [27]. Both circulating leptin and leptin receptors are needed for expression of APOM *in vivo*, although the exact nature of the relationship between obesity and APOM itself is not fully understood [28]. In our study, we have found that expression of APOM is inversely correlated with weight gain in kidney transplant recipients. This result seems to agree with other, plasma-based studies that have found that APOM expression levels are inversely associated with BMI [29], and reduced in those with metabolic syndrome [30], although further work is needed to explore the exact nature of these associations in adipose tissue.

### Future directions

One long-term goal of the parent study is to identify genetic factors that contribute to weight gain in kidney transplant recipients and to test whether those factors could serve as markers for predicting weight gain in this cohort. This analysis should be extended to a larger group that would allow subgroup analysis, for instance between diabetes and weight gain with respect to gene expression changes. Additionally, this pilot study has highlighted the extent of heterogeneity in gene expression profiles among human subjects. In spite of this heterogeneity well-established obesity related genes were found to be associated with weight gain in our study.

This pilot study establishes the feasibility of our methods for identification of candidate genes that may contribute to weight gain in kidney transplant recipients. Future investigations will focus on determining how the genes and pathways identified in this study contribute to weight gain in a much larger kidney transplant population. For instance, we are particularly interested in the alterations of dopaminergic pathways and olfactory pathways related to weight gain. In addition, we plan to compare gene expression profiles in adipose tissue with those in blood to explore a less invasive strategy for predictive expression profiling. It is important to also acknowledge the role of dietary habits and activity levels in preventing obesity following transplantation. While our center encourages our kidney transplant recipients to adhere to a healthy lifestyle (diet and physical activity) and provides dietary counseling when needed, we realize that obesity prevention can be a difficult goal to achieve. As we further refine our list of genes associated with weight gain, we could use this knowledge to better tailor lifestyle changes to prevent the weight gain documented during the first year posttransplant.

### Conclusion

Taken together, the identification of these genes could provide insights into the underlying causes of weight gain in kidney transplant recipients and may lead to discovery of genetic markers for identification of individuals who are at risk of gaining weight after transplantation.

## Materials and Methods

### Ethics Statement

This research was approved by the University of Tennessee Health Science Center Institutional Review Board. Written informed consent was obtained from all subjects.

### Study subjects and data collection

From 2006–2011 investigators from a regional MidSouth transplant institute recruited 153 kidney transplant recipients to participate in a study examining genetic and environmental factors (eg. diet and physical activity) associated with weight gain following transplantation. This paper presents a substudy secondary analysis of data. Institutional Review Board approval was obtained and informed consent was signed by all subjects. All adults approved for kidney transplantation surgery at the transplant institute were eligible for participation in the parent study regardless of race or gender. However, subjects were excluded if prior to kidney transplantation they were taking prednisone or other immunosuppressant therapy. This controlled for any effect of pre-transplantation immunosuppressant therapy on gene expression profiles and baseline weight. Subcutaneous adipose tissue was obtained from a standardized location during the transplantation operation (baseline) and processed for later ribonucleic acid (RNA) transcript analysis. Demographic and clinical data were collected at time of transplantation (baseline) and at 3 months, 6 months, and 12 months.

Thirty subjects who had RNA transcript data, and baseline and 6 months timepoint demographic data, were considered for this substudy. Additional inclusion criteria for the substudy were complete clinical (i.e., immunosuppressant therapy, laboratory values, co-morbidities) and weight data at time of transplantation and at 6 months post-transplant. Of the 30 potential subjects, three had excessive weight loss at 6 months and were excluded from further analysis as it was determined that they experienced an abnormal recovery trajectory. One subject was removed due to technical problems in the RNA measurements as indicated by our quality control procedures. Thus, 26 subjects from the parent study that had a “normal” post-operative course, weight data at baseline and 6 months, and transcript data were included in the secondary analysis. This enabled correlation of baseline transcript expression data with relative weight change over a 6 month period.

Characteristics for the 26 subjects are shown in Table 1. Of the 26 subjects, 24 (92%) were diagnosed with hypertension prior to transplantation. Five of the subjects (19%) had a BMI of greater than 30 kg/m^2^ prior to transplant and thus were classified as obese by the National Institutes for Health guidelines for BMI. All subjects were on similar immunosuppressant therapy protocols during the 6 months following transplantation. Immediately following transplantation, 80% (n = 21) were on 20 milligrams (mg) of prednisone, 8% (n = 2) were on 10 mg of prednisone, 8% (n = 2) were on 15 mg of prednisone, and 4% (n = 1) were on 50 mg of prednisone. At 6 months 92% (n = 24) were on 5 mg of prednisone, and 8% (n = 2) were not taking prednisone.

### RNA expression analysis

Abdominal subcutaneous adipose tissue collection, processing, and storage were described in detail previously [31]. Briefly, specimens were collected intra-operatively from a standardized site in kidney transplant recipients at the time of kidney transplantation. Adipose samples were obtained from the abdominal incision by the transplant surgeon using scalpels. Samples were immediately placed in a Petri dish on ice, moved outside the operating room, cut into 10 sections, placed into individual cryo vials, and then flash frozen in liquid nitrogen. To maintain RNA integrity, the time from collection until freezing was less than two minutes. Specimens flash frozen in liquid nitrogen were then transported to a facility and maintained at −80°C for storage. RNA was isolated using TRizol plus RNeasy Lipid Kit. Quantity of RNA was tested by a NanoDrop® ND-1000 Spectrophotometer (NanoDrop Technologies, Inc., Wilmington, DE). The purity of the RNA was tested by an Agilent 2100 Bioanalyzer (Agilent Technologies, Santa Clara, CA). Finally, RNA expression levels were measured using the Affymetrix Human Gene 1.0 ST GeneChips® (Santa Clara, CA).

qPCR was used to validate the microarray results. RNA was extracted using the Trizol method from additional frozen samples from each of the 26 subjects, and cDNA was made from the Transcriptor First Strand cDNA Synthesis Kit (Roche Applied Science, Indianapolis, IN). qPCR was done using the Taqman® method on the Lightcycler®480. Five of the lowest p-value genes from microarray and two target genes of interest from the microarray findings were used to validate the results [32]. Each gene was normalized to the reference gene using the ΔCt method [33], and then averaged across all 26 subjects to give an average ΔCt. This average ΔCt was then correlated using Pearson product correlation to the microarray results. These results are displayed in Table S3.

### Data Analysis

The relative weight change of the subjects at 6 months after transplantation was regressed on the gene expression levels at baseline while controlling for gender and race. The (marginal) p-values of the regression coefficient associated to the gene expression levels were used to compute q-values using the software available in R [34]. The q-value is the positive false discovery rate (FDR) analog of the p-value and is defined as the expected proportion of false positives among all rejections of the null hypothesis. We found 1936 probes (corresponding to 1553 unique genes) which had a regression p-value<0.05 and a corresponding q-value of 0.48.

Functional analysis of genes was performed using the suite of tools available at WebGestalt (http://bioinfo.vanderbilt.edu/webgestalt/) [35], [36]. Enrichment p-values were calculated for each of the classifications in Gene Ontology (GO) and the Kyoto Encyclopedia of Genes and Genomes (KEGG) using a hypergeometric test with Benjamini-Hochberg FDR correction for multiple testing. Additionally, we used GeneIndexer software (www.computablegenomix.com, Memphis, TN) to rank genes based on relevancy to keyword queries using functional information in Medline abstracts up to March, 2011[37]. GeneIndexer contains over 1.5 million Medline abstracts corresponding to over 21,000 mammalian genes. For this study, GeneIndexer contained abstracts for 1170 (75%) out of 1553 significantly correlated genes. GeneIndexer extracts both explicit and implicit gene-to-keyword relationships from the literature using an information retrieval model called Latent Semantic Indexing [37]. This model ranks genes according to the strength of the association with the keyword query, whereby a score >0.2 typically indicates an explicit association (e.g., the word actually appears in the gene abstracts) and a score between 0.1 and 0.2 typically indicates an implied relationship [37]. A set of 14 neurological and obesity related keyword concepts were manually chosen by the research team and evaluated by clustering using Partek software (St Louis, MO) based on the literature association scores derived by GeneIndexer (Figure S1). We then used Ingenuity software to perform network analysis of the 41 top ranked genes identified by GeneIndexer as having explicit or implicit relationships to obesity keywords. The most prominent network is shown in Figure 4.

## Supporting Information

Table S1 **Expression p-values and q-values for 1936 significant probe sets.** Expression p-values and q-values for 1936 probe sets whose expression levels were significantly (p<0.05, multivariate regression model controlling for race and gender) associated with weight change in 26 kidney transplant recipients.
(XLS)

Table S2 **Top ranked genes associated with keyword concepts.** Genes were ranked according to the number of associations they had with keyword concepts above a score threshold of 0.1. Scores above 0.2 generally suggest an explicit link between the keyword and gene, whereas a score between 0.1 and 0.2 generally suggests an implied relationship between the keyword and gene.
(XLS)

Table S3 **Pearson correlation coefficients between microarray and qPCR.** Pearson correlation coefficients between microarray and qPCR for the top five lowest p-value genes, two target genes of interest, and a reference gene. qPCR was conducted on RNA extracted from adipose tissue samples from the same subjects (using the Trizol method), and cDNA was made using a commercially available kit. qPCR was done using the Taqman® method on the Lightcycler®480.
(XLS)

Figure S1 **Hierarchical clustering of genes based on literature associations with keyword concepts derived by GeneIndexer.** Heatmap representation of similarity values for genes (rows) across keywords (columns), whereby low association score is denoted by green and high score by red.
(TIF)
