# Supplementary figures and images for: Expression Levels of Obesity-Related Genes Are Associated with Weight Change in Kidney Transplant Recipients
Source: PLoS One. 2013 Mar 27;8(3):e59962. doi: 10.1371/journal.pone.0059962 (PMC3609773; doi:10.1371/journal.pone.0059962)

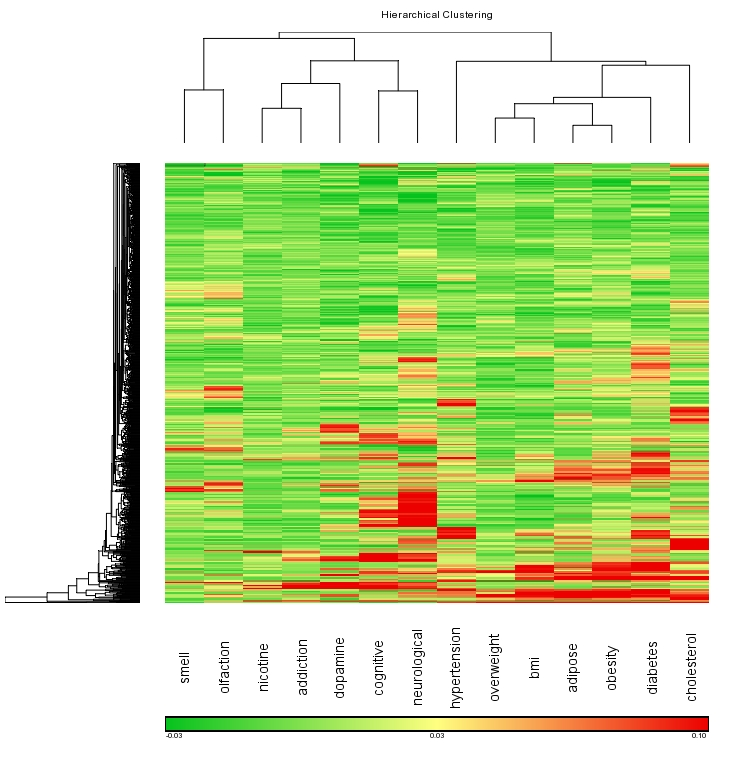

Supplement: Figure S1 — Hierarchical clustering of genes based on literature associations with keyword concepts derived by GeneIndexer. Heatmap representation of similarity values for genes (rows) across keywords (columns), whereby low association score is denoted by green and high score by red. (TIF) [file pone.0059962.s004.tif]
